# Decreased glutathione levels and impaired antioxidant enzyme activities in drug-naive first-episode schizophrenic patients

**DOI:** 10.1186/1471-244X-11-124

**Published:** 2011-08-02

**Authors:** Monia Raffa, Fatma Atig, Ahmed Mhalla, Abdelhamid Kerkeni, Anwar Mechri

**Affiliations:** 1 Research Laboratory of "Trace elements, free radicals and antioxidants", Biophysical Department, Faculty of Medicine, University of Monastir, Avicene street, Monastir 5000, Tunisia; 2 Research Laboratory of "Vulnerability to psychotic disorders", Faculty of Medicine, University of Monastir, Avicene street, Monastir 5000, Tunisia; 3 Department of Psychiatry, University Hospital of Monastir, Avicene street, Monastir 5000, Tunisia

## Abstract

**Background:**

The aim of this study was to determine glutathione levels and antioxidant enzyme activities in the drug-naive first-episode patients with schizophrenia in comparison with healthy control subjects.

**Methods:**

It was a case-controlled study carried on twenty-three patients (20 men and 3 women, mean age = 29.3 ± 7.5 years) recruited in their first-episode of schizophrenia and 40 healthy control subjects (36 men and 9 women, mean age = 29.6 ± 6.2 years). In patients, the blood samples were obtained prior to the initiation of neuroleptic treatments. Glutathione levels: total glutathione (GSHt), reduced glutathione (GSHr) and oxidized glutathione (GSSG) and antioxidant enzyme activities: superoxide dismutase (SOD), glutathione peroxidase (GPx), catalase (CAT) were determined by spectrophotometry.

**Results:**

GSHt and reduced GSHr were significantly lower in patients than in controls, whereas GSSG was significantly higher in patients. GPx activity was significantly higher in patients compared to control subjects. CAT activity was significantly lower in patients, whereas the SOD activity was comparable to that of controls.

**Conclusion:**

This is a report of decreased plasma levels of GSHt and GSHr, and impaired antioxidant enzyme activities in drug-naive first-episode patients with schizophrenia. The GSH deficit seems to be implicated in psychosis, and may be an important indirect biomarker of oxidative stress in schizophrenia early in the course of illness. Finally, our results provide support for further studies of the possible role of antioxidants as neuroprotective therapeutic strategies for schizophrenia from early stages.

## Background

There is strong evidence that oxygen free radicals may play an important role in the pathophysiology of major mental illnesses such as schizophrenia [1,2]. Oxyradicals have a very short life span and usually are inactivated or scavenged by antioxidants before they can inflict damage to lipids, proteins or nucleic acids. The human body has a complex antioxidant defense system (AODS) that includes the antioxidant enzymes: superoxide dismutase (SOD), glutathione peroxidase (GPx) and catalase (CAT). Also more important are the non-enzymatic antioxidants such as glutathione (GSH). Cellular levels of antioxidants respond to levels of oxygen and oxyradicals; which enables cells to defend against increased oxyradical production [3]. If produced in excess, or not removed effectively, oxyradicals result in cellular damage. SOD dismutates superoxide (O_2_^·-^) to yield hydrogen peroxide (H_2_O_2_) and oxygen (O_2_). H_2_O_2 _is not an oxyradical because it does not have an impaired electron, but it must be promptly removed by CAT [3]. Thus, high SOD activity, which results in increased H_2_O_2 _production, must be accompanied by increased GPx and/or CAT activity to limit injury [4]. GPx provides an effective mechanism against cytosolic injury because it eliminates H_2_O_2 _and lipid peroxides (products of ^·^OH mediated peroxidation products) by reduction utilizing GSH [5]. GPx converts peroxides and hydroxyl radicals into nontoxic forms, often with the concomitant oxidation of reduced glutathione (GSHr) into the oxidized form glutathione disulfide (GSSG) and glutathione reductase recycles GSSG to GSH [4]. GSH and other thiol-containing groups also play critical roles as antioxidants. GSH participates in the reduction of oxyradicals and its levels in the brain are high especially during early development [5].

Since oxidative stress is systematic and some of the oxidative products of the brain tissue do end up in the blood, peripheral indices have been accepted to reflect the brain oxidative injury [6]. However, there are conflicting data in the literature on the activities or levels of antioxidant enzymes in patients with schizophrenia. SOD activity in erythrocytes of schizophrenic patients has been reported to be increased [7,8] decreased [9-11] or unchanged [12,13]. GPx activities have been reported to be unchanged [3,9,14-16] but also increased [7,17] or decreased [11,18,19] and CAT activity has been found unchanged [3,13,16] increased [20,21] and decreased [11,15,22]. GSH is the brain's dominant antioxidant implicated in the pathophysiology of schizophrenia [23]. There is a 27% reduction in the cerebrospinal fluid levels of GSH in untreated patients [24] and a similar reduction (41%) in the caudate postmortem of schizophrenic patients [25]. Previous studies recorded a significant decrease in the blood levels of total glutathione (GSHt) [26], of GSHr [27] or of GSHt and GSHr [28] in schizophrenic patients in comparison with controls. Furthermore, increased risk of schizophrenia is associated with polymorphisms of genes associated with GSH synthesis [29,30]. To our knowledge, there are few published studies that have evaluated the antioxidant defense system (AODS) in the blood of first-episode schizophrenic patients (FESP) and most of the studies were conducted on populations of the patients with chronic schizophrenia. Thus, it seemed interesting to consider the medication status and the stage of schizophrenia in the evaluation of the AODS changes that manifest in patients.

The purposes of the present study were (1) to assess whether red blood cell (RBC) SOD, GPx, and CAT activities, plasmatic GSHt, GSHr and GSSG levels were altered in the drug-naive FESP as compared to control subjects, (2) if so, to further test whether altered antioxidant defenses were associated with clinical characteristics of patients.

## Methods

### 1. Subject selection and diagnosis

Twenty-three patients (20 men and 3 women) with a mean age of 29.3 ± 7.4 years (range, 19-45) were recruited in their first-episode of schizophrenia from consecutive admissions at the psychiatric department of the University Hospital of Monastir. They provisionally had DSM-IV-TR criteria for schizophrenia (n = 9) and schizophreniform disorder (n = 14) based on the Structured Clinical Interview for DSM-IV-TR [31]. Diagnosis was reconfirmed at 6 months by consensus. Healthy control subjects (n = 45, 36 men and 9 women with a mean age of 29.6 ± 6.2 years, range, 22-47) were recruited from blood donors in the blood center of the University Hospital of Monastir. Their current mental status and personal or family history of any mental disorder was assessed by unstructured interviews.

The exclusion criteria considered for the two groups were the same and included the following parameters: seizure disorders, head injury with loss of consciousness, dependence on alcohol and other substances (except dependence on tobacco), vitamin supplementation followed for 6 months prior to inclusion in the study, and denial to take part in the present study. Additional exclusion criteria for the control subjects included personal or family history of psychosis. All subjects signed informed consent after a full explanation of the study. The study was approved by the Local Ethic Committee of the University Hospital of Monastir. The demographic and clinical characteristics of the FESP and the control subjects were summarized in Table 1. Age and gender distribution of the subjects and smoking habit did not differ between patients and controls (Table 1).

**Table 1:** Demographic and clinical features of the patients and the controls

Demographic and clinical features	Patients (N = 23)	Controls (N = 45)	p-values
**Age (mean ± SD) (years)** **Min-Max**	**29.3 ± 7.5** **19-45**	**29.6 ± 6.2** **22-47**	**0.995**
Gender (male/female)	20/3	36/9	0.477
Smokers (%)	56.5	46.7	0.44
SAPS score (mean ± SD)	25.4 ± 12.2	-	
**SANS score (mean ± SD)**	**29.5 ± 16.5**	**-**	

Max: maximum, Min: minimum, SAPS: Scale for assessment of positive symptoms, SANS: Scale for assessment of negative symptoms, SD: Standard deviation.

### 2. Biochemical procedures

Five milliliters of blood was drawn from control subjects and FESP by simple venipuncture between 7.00 and 9.00 a.m. after overnight fasting and tobacco abstinence for > 12 h. For the patients the blood samples were obtained prior to the initiation of neuroleptic treatments. The samples were centrifuged for 10 min at 3500 rpm. Plasma RBCs were then separated, aliquoted and stored at -80°C until analysis. For all samples, each evaluated parameter was assayed in duplicate. Throughout the investigations the biochemical assays were conducted blind of the available clinical information. The total SOD activities were determined using pyrogallol as substrate by the method of Marklund and Marklund [32]. This method is based on pyrogallol oxidation by the superoxide anion (O_2_^-^) and its dismutation by SOD. One unit (U) of total SOD is defined as the amount of enzyme required to inhibit the rate of pyrogallol autoxidation by 50%. GPx activity was assayed by the subsequent oxidation of NADPH at 240 nm with t-buthyl-hydroperoxide as substrate [33]. While, CAT activity was determined using the method described by Beers and Sizer [34] by measuring hydrogen peroxide decomposition at 240 nm. CAT units (U/mg hemoglobin) were determined as mmol of H_2_O_2 _consumed/s/mg hemoglobin. The total hemoglobin content was measured as cyanmethaemogobine using the drabkin method. The glutathione levels were measured spectrophotometrically in deproteinized blood samples, by the method of Akerboom and Sies [35], using 5,5 dithiobis (2-nitrobenzoic acid). Absorbance values were compared with standard curves generated from known amounts of GSH standards.

### 3. Psychopathological assessment

Patients were rated for psychopathology using the Scale for the Assessment of Positive Symptoms (SAPS) and the Scale for the Assessment of Negative Symptoms (SANS) [36]. The assessments were carried out by trained psychologists.

### 4. Statistical analysis

The Windows computing program Statistical Package for the Social Sciences SPSS 10.0 [37] was used for analyzing the data. The obtained data was presented as mean values ± standard deviation (SD) and were analyzed using nonparametric statistics. Specifically, between groups, comparisons were examined using Mann-Whitney tests. The adjusted analysis of variance (ANOVA) was used in case the parameters of age, gender, and the smoking habit of the subject were found to have some effects on the levels of glutathione and the activities of the antioxidant enzymes. Finally, the correlations existing between the antioxidant systems and the SAPS and SANS scores in our patients were calculated by using Spearman correlation coefficients. The differences were considered significant at values of p ≤ 0.05.

## Results

### 1. Antioxidant enzyme activities and glutathione levels

There was no significant correlation between age and AODS included enzyme activities and glutathione levels, in patients or healthy controls. Similarly, there was no effect of gender on AODS, even though the activities of CAT and GPx were found to be affected by the smoking habit of patients (U de Mann-Whitney = 24, p = 0.02 and U de Mann-Whitney = 25, p = 0.04 respectively). Thus, the differences that appeared in the activities of RBC antioxidant CAT and GPx activities were tested by executing an ANOVA adjusted by the smoking habit of the subjects.

In this study, no significant difference was observed in the RBC SOD activity between FESP and control subjects. RBC GPx activity was significantly higher in patients than in healthy controls with 68.6 ± 13.5 *vs*. 41.1 ± 26.3 U/g Hb (F_1-64 _= 31.8, p < 0.001), but RBC CAT activity was significantly lower in patients than in controls with 212.2 ± 36.9 *vs*. 284.6 ± 88.2 U/g Hb (F_1-64 _= 14.8, p < 0.001) (Table 2). As shown in Table 3, GSHt and GSHr levels were significantly lower in FESP than in control group with values: 560.4 ± 123.6 μmol/l *vs*. 759.5 ± 260.9 μmol/l (U de Mann-Whitney = 214, p = 0.001) for GSHt and 512.1 ± 117.7 μmol/l *vs*.732.2 ± 274.6 μmol/l (U de Mann-Whitney = 185, p < 0.001) for GSHr, respectively. Whereas, GSSG was significantly higher in patients than in controls (U de Mann-Whitney = 270, p = 0.013) (Table 3). Additionally, we have not found any significant differences between schizophreniform (n = 14) and schizophrenic patients (n = 9) in terms of antioxidant enzyme activities and glutathione levels.

**Table 2:** The activities of the RBC antioxidant enzymes in the study groups

Antioxidant enzymes	Patients (N = 23)	Controls (N = 45)	p-values
**SOD (U/mg Hb)**			
**Mean ± SD**	**2.2 ± 0.6**	**2.4 ± 0.6**	**0.2**
**Min-Max**	**1.5-3.8**	**1.7-4.6**	
GPx (U/gHb)			
Mean ± SD	68.6 ± 13.5	41.1 ± 26.3	< 0.001*
Min-Max	28.2-122.6	22.9-70.7	
**CAT (U/gHb)**			
**Mean ± SD**	**212.2 ± 36.9**	**284.6 ± 88.2**	< 0.001*
**Min-Max**	**157.3-282.5**	**100.3-499.3**	

CAT: catalase, GPx: glutathione peroxidase, Max: maximum, Min: minimum, RBC: red blood cell, SD: standard deviation, SOD: superoxide dismutase.

*Adjusted by smoking habit

**Table 3:** Glutathione levels in the study group

Glutathione levels	Patients (N = 23)	Controls (N = 45)	p-values
**GSHt (μmol/l)**			
**Mean ± SD**	**560.4 ± 123.6**	**759.5 ± 260.9**	**0.001**
**Min-max**	**402.9-821.2**	**430.6-1537**	
GSHr (μmol/l)			
Mean ± SD	512.1 ± 117.7	732.2 ± 274.6	< 0.001
Min-max	402.9-821.2	374.8-1514.8	
**GSSG (μmol/l)**			
**Mean ± SD**	**47.8 ± 18.1**	**35.4 ± 20.3**	**0.013**
**Min-max**	**10.6-76.6**	**6.5-73.2**	

GSHr: reduced glutathione, GSHt: total glutathione, GSSG: oxidized glutathione, Max: maximum, Min: minimum, SD: standard deviation.

### 2. Correlations between the antioxidant system and the SAPS and SANS scores

There was a positive correlation between the score of SAPS and the levels of tGSH and rGSH (r = 0.50, p = 0.04; r = 0.51, p = 0.03, respectively). However, antioxidant enzyme activities were not significantly correlated to SAPS and SANS scores. Correlation coefficients between SAPS, SOD, GPx and CAT were as follows (r = -0.34; r = -0.11 and r = -0.11; respectively). Correlation coefficients between SANS and SOD, GPx and CAT were as follows (r = -0.12; r = -0.15 and r = -0.12; respectively) (Table 4).

**Table 4:** The value of Spearman correlation coefficients calculated between the antioxidant system and the SAPS and SANS scores in drug free FESP

Antioxidant system	SAPS scores	SANS scores
**Total glutathione**	0.50*	**-0.02**
Reduced glutathione	0.51*	-0.05
**Oxidized glutathione**	**0.16**	**0.17**
Glutathione peroxidase	-0.34	-0.12
**Superoxide dismutase**	**-0.11**	**-0.15**
Catalase	-0.13	-0.22

FESP: first-episode schizophrenic patients, SAPS: Scale for assessment of positive symptoms, SANS: Scale for assessment of negative symptoms.

*p < 0.05

## Discussion

The key results of the present study were: (1) the levels of GSHt and GSHr significantly decreased in the drug-naïve FESP in comparison with the control subjects. (2) the group of FESP revealed an increased activity of GPx and a decreased activity of CAT in RBCs. (3) a positive correlation exists between the score of SAPS and the levels of GSHt and GSHr. The findings of this study indicate that some FESP may be poorly equipped to deal with oxidative stress due to impaired antioxidant defenses. Moreover, oxidative stress might play a role in the brain's developmental and maturational processes in the pathogenic cascade of schizophrenia. The findings reported above suggest such a possibility and call for more systematic research on the role of oxidative stress in schizophrenia.

The detailed neurochemical mechanisms underlying the pathophysiology of schizophrenia are not clearly understood. There has been accumulating evidence supporting the involvement of oxidative stress in the pathophysiology of this disease [2]. Prabakaran et al. [38] reported that transcript, protein and metabolite alterations are associated with the mitochondrial function and oxidative stress in the cortex, the liver and the RBCs of schizophrenic patients. The antioxidant system eliminates reactive oxygen species to maintain a reduced environment in cells through enzymatic or non-enzymatic approaches. The most studied antioxidants are the SOD, GPx and CAT enzymes. Notably, CAT and SOD, acting in concert with GPx, constitute the major defense or primary antioxidant enzymes against superoxide radicals [39]. However, it is important to underline the contradictions and the controversial outcomes found in the literature. In fact, these differences can be due to several variables among which are inclusion and exclusion criteria for patient selection, analytical methodologies, testing materials (blood cells vs. plasma or serum), exposure to medication (naïve *vs*. drug withdrawal *vs*. medicated), stages of the disease (acute *vs*. chronic or active *vs*. remission phase), lifestyle or dietary pattern, and the patient's origin. The main reason for the difference between the current study and those previously reported [3,24,40], is likely to be the early stage of illness of our patient sample. Also, our study focused on drug-naïve FESP to show whether the oxidative disturbances which occur during the course of the disease can be related to the degenerative process linked to the symptoms and/or treatment, or rather related to schizophrenia and appear at an early stage of the disease. Studies comparing first-episode and chronic schizophrenic patients would be necessary to further investigate a stage-specific change in AODS in schizophrenia.

In our research, we tried to explore the activities of SOD, GPx and CAT in the RBCs of our collected samples of patients and controls. In the present study, we found that the activity of SOD, a key enzyme in the endogenous antioxidant defense pathways, did not differ between the FESP and controls. Similarly, Mico et al. [41] found no significant difference in SOD activity between early-onset first-psychosis group and the control group. Other studies have reported lower SOD activity in neuroleptic-naïve FESP [3]. High levels of blood SOD were reported in neuroleptic-naive schizophrenic patients [8] and higher activities of SOD in neuroleptic-free schizophrenic patients in comparison with the schizophrenic patients treated with haloperidol [13]. However, in our study, the levels of GPx were significantly higher in the FESP than those in control subjects. The same result observed in the early-onset first-episode psychosis by Mico et al [41]. Yao et al. [13] showed also a significant increase in GPx activity in drug-free schizophrenic patients compared to treated ones. In this case, increased GPx antioxidant activity may reflect a preceding cellular oxidative stress or serve as a compensatory mechanism. Interestingly, the CAT activity was significantly lower in the RBCs of drug-naïve FESP than that in control group. Other studies [3,41] showed no significant difference in CAT activity between early-onset first-psychosis patients and the control subjects. Raffa et al. [28] found a significant decrease of CAT activity in neuroleptic-free schizophrenic patients.

On the other hand, it is likely that oxidative stress injury was due to an impaired antioxidant defense in early stage of schizophrenia. Furthermore, because of their impaired antioxidant defense, some patients might be vulnerable to oxidative injury in spite of their normal oxyradical production [42]. In recent decades, biochemical studies have increasingly more often focused on the role of free radicals in the pathogenic of neuropsychiatric diseases such as schizophrenia [43,44]. In addition to the impaired antioxidant enzyme activities, we also found a decreased plasma GSHt and GSHr levels in the drug-naïve FESP. Previous studies recorded a significant decrease in the RBC levels of GSHt [26] or of GSHr [27] in schizophrenic patients in comparison with the controls. Plasmatic GSH level was significantly lower in the FESP [41], magnetic resonance spectroscopy studies have shown that levels of GSH were reduced by 52% in the prefrontal cortex and by 27% in the cerebrospinal fluid of drug-naïve schizophrenic patients [24]. However, a spectroscopy study showed that patients with first-episode psychosis had a higher concentration of GSH in the medial temporal lobe than control group [23]. Anomalies in GSH metabolism were also supported by the low expression of the gene of the key GSH-synthesizing enzyme, glutamate cysteine ligase modifier subunit, in patient fibroblasts [30]. The GSH deficit found in this study and in previous reports [18,25,45,46] may be involved in membrane peroxidation and microlesions related to dopamine, which seem to be increased in psychosis, and suggest that GSH may be a possible indirect indicator of damage in neuronal membranes [47,48]. This study of the drug-naïve FESP also suggests that the deficit in GSH may underlie the pathophysiology of the disease and is not a consequence of treatment. The converging data in literature, in agreement with our results in FESP, indicate that psychosis is associated with an important brain glutathione deficit. In fact, it could be hypothesized that different etiological mechanisms converge into precipitating a first psychotic episode in individuals with a limited GSH synthesis capacity, after which the psychotic episode develops into a degenerating condition that we call schizophrenia. This could be tested by analyzing glutathione in high-risk populations that are subsequently followed up.

In our patient group, using the SAPS, the presence of positive symptoms was associated with higher levels of GSHt and GSHr. Positive symptoms are associated with subcortical dopamine hyperactivity in schizophrenia [49]. Several studies have revealed that catecholamines, especially dopamine, are associated with free radical generation [47-49]. As suggested in the present study, this may serve as a compensatory or protective mechanism employed to neutralize oxidative stress produced from presumably supra-physiological prefrontal dopamine. Therefore, the hyperdopaminergic state in schizophrenia, induced by still unknown mechanisms, may explain the positive association between positive symptoms and GSH levels in the present study. Although, we should be cautious, our findings support the possibility of using peripheral markers of oxidative and antioxidative system in FESP, taking into account the special sensitivity of the brain to oxidative damage [50].

This study has some limitations. Diagnostic groups were relatively small, and it was difficult to establish in advance a sample size to perform the data analyses because of the paucity of studies with similar design characteristics. A second limitation was the used samples were limited to blood ones. Because the data presents changes in peripheral blood, further work is needed to determine if such changes adequately reflect changes in the brain. Although, recent findings have identified a genetic origin of GSH deficit, which results in the impairment of the GSH synthesis in patients diagnosed with schizophrenia [51]. The decreased levels of GSH and/or the activities of antioxidant enzymes in the peripheral blood of the patients may indicate the occurrence of a systematic reaction that may cause oxidative stress in the brain of schizophrenic patients as is the case in other disorders of the central nervous system [52]. The strengths of the study are the uniformity in age with first-episode and drug-naïve of schizophrenic patients, and the existence of a control group.

## Conclusion

In summary, our study shows that there is impairment in the AODS in drug-naive FESP. GSH deficit seems to be implicated in psychosis, and may be an important indirect biomarker of oxidative stress in first-episode of schizophrenia. Our results provide support for further studies of the possible role of antioxidants as neuroprotective therapeutic strategies for schizophrenia from early stages [6]. Data from the longitudinal study will clarify the possible utility of peripheral markers of oxidative stress as prognostic factors and the effect of neuroleptic drugs on oxidative stress.

## Abbreviations

(AODS): Antioxidant defense system; (CAT): Catalase; (DSM-IV-TR): Diagnostic and statistical manual of mental disorders; (FESP): First-episode-schizophrenic-patients; (GPx): Glutathione peroxidise; (GSHr): Reduced glutathione; (GSHt): Total glutathione; (GSSG): Oxidized glutathione; (Max): Maximum; (Min): Minimum; (RBC): Red blood cell; (SANS): Scale for assessment of negative symptoms; (SAPS): Scale for assessment of positive symptoms; (SD): Standard deviation; (SOD): Superoxide dismutase.

## Competing interests

The authors declare that they have no competing interests.

## Authors' contributions

All the authors made substantial contributions to the design and conception of the study. Particularly, MR wrote the manuscript, contributed to the analysis and interpretation of the data. AM conceived of the study, and participated in its design and coordination and helped to draft the manuscript. AK contributed to the development of the protocol and study instruments. All the authors have been involved in drafting and revising the manuscript, have read, and approved the final manuscript.

## Pre-publication history

The pre-publication history for this paper can be accessed here:

http://www.biomedcentral.com/1471-244X/11/124/prepub
